# Cross-domain neurobiology data integration and exploration

**DOI:** 10.1186/1471-2164-11-S3-S6

**Published:** 2010-12-01

**Authors:** Weijian Xuan, Manhong Dai, Josh Buckner, Barbara Mirel, Jean Song, Brian Athey, Stanley J Watson, Fan Meng

**Affiliations:** 1 Psychiatry Department and Molecular and Behavioral Neuroscience Institute, University of Michigan, USA; 2 School of Education, University of Michigan, USA; 3 Health Science Libraries, University of Michigan, USA; 4 National Center for Integrative Biomedical Informatics, University of Michigan, USA

## Abstract

**Background:**

Understanding the biomedical implications of data from high throughput experiments requires solutions for effective cross-scale and cross-domain data exploration. However, existing solutions do not provide sufficient support for linking molecular level data to neuroanatomical structures, which is critical for understanding high level neurobiological functions.

**Results:**

Our work integrates molecular level data with high level biological functions and we present results using anatomical structure as a scaffold. Our solution also allows the sharing of intermediate data exploration results with other web applications, greatly increasing the power of cross-domain data exploration and mining.

**Conclusions:**

The Flex-based PubAnatomy web application we developed enables highly interactive visual exploration of literature and experimental data for understanding the relationships between molecular level changes, pathways, brain circuits and pathophysiological processes. The prototype of PubAnatomy is freely accessible at: [http://brainarray.mbni.med.umich.edu/Brainarray/prototype/PubAnatomy]

## Background

Data driven research using high throughput experimental approaches, such as microarray, genome-wide association study, deep sequencing and structural and functional imaging, is now one of the major driving forces for the advancement of biomedical research. The prevalence of high throughput experiments also brings significant challenges in the analysis and the understanding of data. Frequently researchers only publish and interpret a very small fraction of high throughput data, focusing on statistically most significant data points that they can establish plausible link with the target biological processes. Achieve comprehensive understanding of the high throughput data is very difficult since most of the data points are not in the areas that the researchers are familiar with. Linking unfamiliar genes, SNPs, sequences and voxels to specific pathophysiological processes necessitates cross-domain data integration across different scales.

There are several major challenges inherent to cross-domain data integration and mining. The first challenge is to link concepts from different domains. Most public and commercial databases and programs deal with only closely related domains. For example, various molecular biology databases presented in the annual database issue of the journal Nucleic Acid Research deal only with aspects of gene/protein structure and functions. There are a number of databases that contain cross-domain information such as disease-gene or gene-MeSH term relationships [1]. However, these databases are not usually suitable for in-depth data exploration and mining due to their limited scope.

True cross-domain and cross-scale databases designed for deep data exploration and mining are rare. The data integration effort by the Biomedical Informatics Research Network (BIRN) is an exceptional example [2,3]. Nonetheless, BIRN's core data integration and exploration solutions are only available to BIRN related researchers. Since brain imaging data analysis for advancing diagnosis and treatment of diseases is the original purpose of BIRN, their solutions do not contain tight integrations with molecular level data. Molecular level data is now, arguably, the major source of high throughput data in terms of the number of researchers involved and publications.

Commercial solutions from companies such as Ingenuity (Ingenuity Pathway Analysis), GeneGo (MetaCore) and Genomatix (GenomatixSuite and BiblioSphere) provide excellent integration different aspects of molecular level events. However, while the commercial solutions' reliance on expert curation enhances the accuracy of their knowledge bases, it also places significant limitations on their coverage of existing biomedical knowledge. In fact, while these solutions are excellent for exploring molecular level events, their gene-centered nature and the significant lack of higher level conceptual relationships (e.g., relationship between an organ structure and a disease) severely limits their usefulness in cross-domain knowledge exploration and mining. Solutions that capable of integrating the full spectrum of data from molecular to organism levels are highly desirable.

Another challenge of cross-domain data integration and mining is that, even if concepts from different domains are integrated, it is not easy to present them in a way that facilitates both effective overviews and in-depth investigation. Lists of results as well as graphic presentations often fall short of promoting efficient browsing and exploratory analysis. For example, although the Medline database is arguably the most comprehensive cross-domain knowledge base, most Medline search engines present search results as linear lists of returned records. It is hard to navigate long lists and they do not facilitate discerning novel conceptual relationships across different records, either. Even when retrieved records are presented in sortable table format (e.g., our GeneInfoMiner [4] and MarkerInfoFinder [5]) or in tree format after mapping them to ontology/MeSH terms (e.g., GoPubMed [6] and our PubOnto [7]), it is difficult to integrate different records for new relationship discovery.

Graphical search and analysis solutions attempt to help biomedical researchers to identify conceptual relationships among different records [8-14]. Medline search solutions such as PubGene [11], ALIBABA [9], botXminer [10], and Chilibot [15] strive to help users to perceive conceptual relationships more readily through network graphs. Another popular graphic Medline solution, RefViz, provides a “galaxy” view based on similarity-based clustering [16] but cannot provide real time clustering due to computing speed limitation on typical desktop computers. These solutions are more suitable for knowledge exploration than list-style displays since they enable researchers to grasp some complex biomedical conceptual relationships at a glance and aid them in identifying related but often unexpected gene-oriented conceptual relationships pertaining to the search topic [9]. Nonetheless, apprehending meaning while browsing and exploring in conceptual networks can quickly become difficult as the number of concepts increases beyond 50 or so. Thus users merely see a “spaghetti ball” of many-to-many associations. More biologically relevant visualization solutions with high computation efficiency are needed.

The third major issue in existing data integration and mining solutions is most of them has a closed architecture. Few publicly available solutions allow users to utilize their own data and/or analysis algorithms. Current graphic Medline search engines only allow limited display parameter adjustment for built-in layouts, and it is impossible for third party developers or users to add new display methods for different search or exploration requirements. In addition, most solutions cannot interoperate with each other, either. It is not possible for researchers to take advantage of complementary solutions seamlessly during data exploration and mining processes. An open solution that enables other researchers and developers to integrate new data and functions will be very important.

Here we present our work that aims to address some of the issues described above by focusing on cross domain data mining requirements in the area of neurobiology. Our solution uses the Medline database as the backbone knowledge base but integrates several types of important molecular and structural level data. The integration of cross-domain data outside of the Medline database is achieved through a highly efficient custom biomedical concept identification engine. We choose anatomical structure as the framework for presenting an overview of search results as well as the starting point for more detailed investigations. Our solution adopts an open architecture and enables interoperability with other web applications based on a general schema. Our prototype solution is called PubAnatomy and it is developed on the Adobe Flex platform for highly interactive visual exploration.

## Methods

### A. Backbone knowledge base selection

Given the cost and the limitation of expert-based curation, we decided to use the Medline database as the backbone database for cross-domain and cross-scale data integration. The Medline database is without a doubt the foremost biomedical knowledge base. It has the most complete coverage of all areas of biomedical research among the existing databases. It is essential to most, if not all, biomedical researchers for exploring relevant topics and understanding the biological implications of their own data. Inherent conceptual relationships in Medline abstracts, titles and MeSH terms can be directly used for linking and understanding concepts from different biological scales and biomedical research domains. This is a critical advantage that is very hard to match by an expert-curated system.

### B. Identification of biomedical concepts in free text

Although Medline database contains comprehensive information, it is essential to identify all useful biomedical concepts in Medline title and abstracts for the purpose of cross-domain data integration and computer-based analysis. While data from closely related biomedical research domains can often be linked to well defined molecular IDs (e.g., gene ID), structure IDs (e.g., anatomical structure) or spatial coordinates (genomic location or 3D anatomical locations), cross domain data integration frequently has to rely on the matching of different free text strings representing the same concepts or identifying relationships between different concepts. It is essential that the free text strings in the Medline database can be converted into computable forms for computer-based mining and more effective data presentation.

In collaboration with the National Center for Biomedical Ontologies (NCBO), we developed a highly efficient and flexible free text to biomedical concept mapping solution called mgrep. It has three major advantages over the publically available MetaMap Transfer (MMTx) program developed by the National Library of Medicine [http://mmtx.nlm.nih.gov]: 1) mgrep records the location of each matched concept in the original text, which is critical for in-depth text mining 2) mgrep is about 2 orders of magnitude faster than MMTx. It enables us to process the Medline dataset frequently to keep up with updates from various controlled vocabulary sources as well as Medline on a single dual opteron server 3) MMTx is designed to work with concepts in the Unified Medical Language System alone but it is very straightforward for our solution to include and manage additional vocabularies, such as chemical compound names and ontologies in the Open Biomedical Ontologies (OBO) system [17].

In comparative analysis of concept mapping results using all of the concepts in UMLS and 10,000 Medline sentences as input, mgrep can identify about 95% of best match concepts that MMTx can find. We believe this level of sensitivity is sufficient for data integration and exploration purposes. Although the details of our work in this area have yet to be published, a fully functional implementation of mgrep for on-the-fly free text to OBO ontology mapping is available at [http://bioontology.org/tools/oba.html]. Using mgep, we are able to identify free text strings to unique concepts in full UMLS and 10 or so OBO ontologies related to anatomy, disease, environment, and chemicals in the Medline database. We also identifies concepts related to genes, genetic markers and cytobands using entity recognition engines developed in earlier work [4,18,19]. The transformation of highly variable free text strings to computable unique biomedical concepts as well as the ability to identify conceptual relationships based on UMLS and ontologies provide the foundation for cross domain data integration in our database.

### C. Integrating data from external sources

The mgrep program's ability to map free text strings to unique biomedical concepts provides unlimited data integration possibilities. For example, even in the absence of common molecular and structure IDs, mgrep can be applied to free text fields in a database to link together information from different domains. While such direct concept mapping will not always be correct, we believe the benefits outweigh the shortcomings. Our main goal is to facilitate novel hypothesis development by present researchers with a more comprehensive view of related issues rather than providing 100% accurate conceptual relationships.

Naturally, our system supports integration of data outside of the Medline database. Our system's data integration is based on widely used IDs and coordinates such as Gene IDs and genomic locations. Since our prototype is focusing on neurobiological problems, we also enable the integration of data based on Allen Brain Atlas structure coordinates.

For example, in order to link Medline records to individual brain structure names in the Allen Brain Atlas, we downloaded canonical mouse and rat brain structure nomenclatures in four brain atlases from the BrainInfo website [http://braininfo.rprc.washington.edu/Nnont.aspx]. The same page provides a mapping of each term to the NeuroName2002 ontology for human and macaque neuroanatomy. We combined all distinct brain structure name text strings from different atlases and NeuroName into a list of 13233 test strings representing various brain structures. Since one of the atlases annotated by the BrainInfo website is the Dong atlas used by the Allen Brain Project, we are able to map all text strings from other atlases and NeuroName to brain structure terms used by the Allen Brain Atlas based on the NeuroName annotation provided by the BrainInfo project.

To reduce false positives in the brain anatomical structure mapping, we exclude abbreviations and require each structure term to be at least 5 characters long. Then we use the lvg program to generate common lexical variations of each text string; we eliminate high frequency words without distinctive meaning, e.g. "the" and "of", that we identified in an earlier full Medline text analysis; and we generate word order permutations. Next, using mgrep, all string variations are mapped to the full Medline abstracts and titles (again discarding meaningless high frequency words). To further increase the sensitivity of identifying Medline records related to brain structures, we also included records with MeSH terms that can be directly mapped to structure terms in the Allen Brain Atlas. The combination of text-string and MeSH based mapping leads to close to 1 million Medline abstracts that can be linked to structure names in the Allen Brain Atlas for the December, 2008 download of the Medline database.

### D. Identify potentially important conceptual relationships

Our extensive concept mappings of the full Medline database allow us to associate pairs of over 1 million unique biomedical concepts from different biomedical research domains based on concept co-occurrence at either the abstract or the sentence level. However, such concept co-occurrence association may lead to a large number of false relationships that will reduce the efficiency of data exploration and mining. However, short of expert curation, there is no satisfactory way to extract accurate conceptual relationships with decent sensitivity. While significant progress has been made in the area of natural language processing [20], the best solutions for extracting conceptual relationships are still not ideal.

Since our goal is to help researchers to mine data more effectively, we decided to compare the level/frequency of a concept in a given context (e.g., Medline records returned by a keyword search) to the concepts level/frequency in the full database (e.g., Medline) to rank the concept's importance in the given context. The underlying assumption is that the concepts ranked most significant by this approach are the concepts most likely to have meaningful relationships to the query term(s). Two main advantages of this approach is it is computationally efficient and this approach can be applied to different data types.

For example, we use this approach to identify the most significant disease terms from a list of Medline records in the current solution as well as in our earlier PubOnto web application [7]. Briefly, based on disease concept mapping and MeSH term annotation of each Medline abstract, we are able to pre-calculate the overall frequency of each disease-related concept in the Medline database. The frequency of disease terms in each set of returned Medline records, regardless of what search terms and filters are used, can be ranked against their background values using a number of different ways on-the-fly. Consequently, users can easily identify the most significant concepts associated with the search results, out of a very large number of concepts that have abstract or sentence level co-occurrence, for further exploration. While there is no guarantee that such a simple statistical approach can always identify the most meaningful conceptual relationships, it provides a valuable starting point for data mining.

We also applied the same approach to rank genes that are expressed in individual brain structures based on the voxel level gene expression data from the Allen Brain Atlas [21]. Since the Allen Brain Atlas provides 200 micron voxel level expression data for around 20,000 genes in each voxel, methods to select the most relevant genes are necessary for more effective exploration of functional relationships between genes and brain structures. One of the ranking methods we included in the current solution is the ratio of the average gene expression level derived from all voxels belonging to a brain structure and the average gene expression level based on all voxels in the whole brain. This method turns out to be quite effective in identifying genes that are highly expressed in distinct brain regions. For example, the top 20 genes in each brain region identified by this simple method show on the average of 21-fold higher expression level in a specific brain region than their average expression level in the whole brain. Consequently, researchers can easily identify genes that are most significantly expressed in a brain region to further explore their functional relevance.

### E. Use anatomical structure as a scaffold

Identification of brain anatomical structure concepts in Medline records not only facilitates the integration of data with brain structures, but also enables the use of the Allen Brain Atlas as both an overview of the data and a starting point for data exploration in a relevant biological context. There are several reasons for selecting brain anatomical structure as the anchor for data presentation and exploration: anatomical structure is a biologically meaningful way for integrating cross-domain data and literature since the majority of pathophysiological processes in the brain can be linked to specific brain structures; hierarchical and brain circuit level relationships among different brain structures (important for detailed data exploration but not obvious for most molecular biologists) are easily presented in brain anatomy; the presentation of anatomical structure at the level of major brain nuclei is not as overwhelming as complex network graph; and the fixed location of each brain structure at a specific brain section plane allows quick identification of relevant content.

Without a doubt, alternative perspectives of the same data are needed. In PubAnatomy, we included a gene network view and various list views in our user interface. These views are described in the RESULT section. We also incorporate a new solution aiming at taking advantage of compatible external applications for more effective data exploration and it will be described in the next section.

### F. Interoperability with other applications

In order to share data among multiple applications, we design a simple and effective schema to accommodate the following requirements: data generation, individual or group access permission, diversified data fields, central dataset registry, and incremental updates of data sets.

Our schema involves a set of five relational database tables: 1) User account table: maintains the groups that users belongs to; 2) Share permission table: the original creator of a data set can set the access permission of the data set, including individual accounts, group accounts, and public; 3) Dataset definition table: keeps the title and field names of each set; 4) Data storage table: the ultimate table for storing data of each set; 5) Update history table: tracks the history of set changes, including the application, type of change (e.g., create/update/delete), and the parameters used to determine the change of a data set.

There are other alternatives in implementing the data sharing and interoperability. Typically, they are session data sharing and web service frameworks. The session- based data sharing’s initial simplicity will be outweighed by difficulties in cross domain data exchange due to various firewalls settings, organizational security settings and browser requirements. The pure web service approach is not satisfactory since function changes need to be coordinated by different groups. The above data sharing schema is more stable due to its ability to accommodate future function changes once the same schema is adopted across groups.

## Results

### A. Overall Architecture

**Figure 1:**
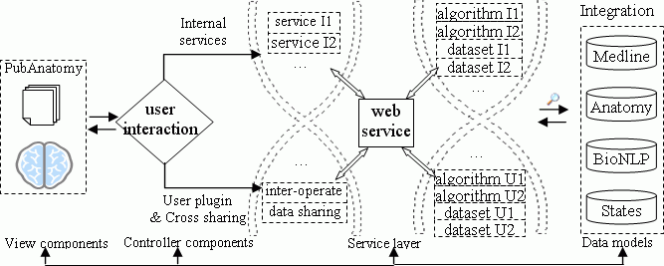
**PubAnatomy architecture** The component-based open architecture of PubAnatomy. PubAnatomy is developed based on Adobe’s latest Flex 3.0 platform. It follows Model-View-Controller-Service design pattern. It allows us to build a highly interactive user interface that is compatible in virtually all major browsers.

The high level architecture of PubAnatomy follows the Model-View-Controller- Service design pattern, as shown in Figure 1. The view component (UI) is a Flex- based Rich Internet application compatible with virtually all major browsers. The UI is further divided into a series of views that each is a reusable component. The controller component handles user interactions (e.g. keyword search, gene expression heatmap drawing) and associated events, and call services to analyze and exchange data. The service layer provides the bridge between UI and the PubAnatomy backend or external databases. There are already over 30 web services developed for PubAnatomy. Except services dedicated to providing UI information, the web services can also serve other programs. The data models maintain datasets and stores the state of the user exploration in PubAnatomy. Overall, the decoupling of interaction, UI design, data models and analysis enables us to handle nonlinear and complex exploration process.

**Figure 2:**
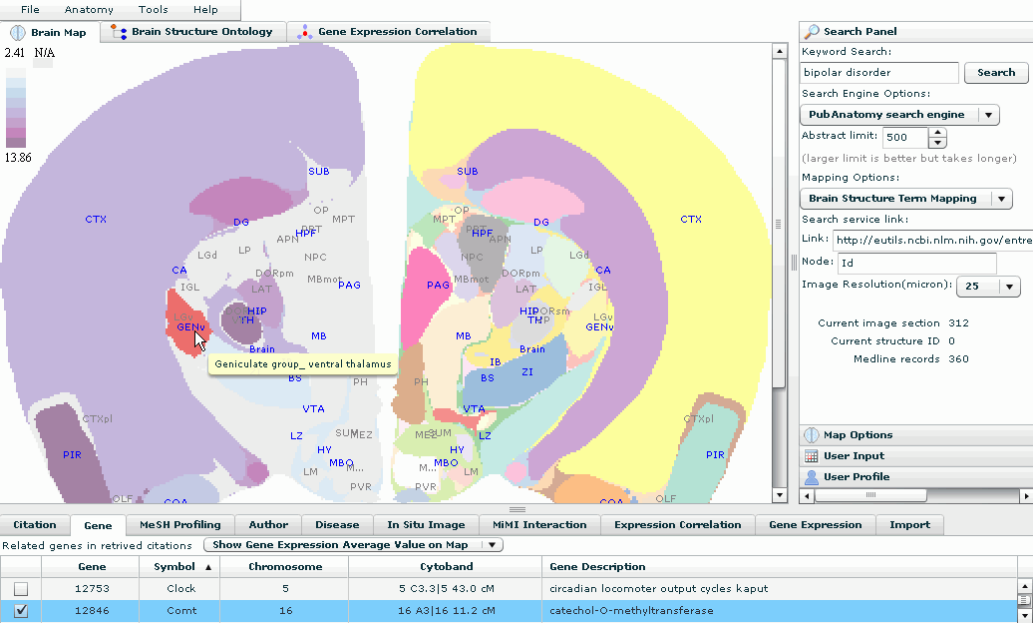
**PubAnatomy user interface overview** PubAnatomy provides different view and data tables for filtering and sense making. Its UI has three major components: 1) graphic views on the main window provide data overview (such as gene expression data display) and starting points for data exploration 2) tabulated data tabs in the bottom contain information relevant to the current view and selection, such as current brain structure, citation set, selected genes, etc.; 3) Tabs and menus on top of the main window are for selecting parameters and initiating analysis. The right panel contains search functions, user input and user history management.

### B. Data exploration and analysis functions

**Figure 3:**
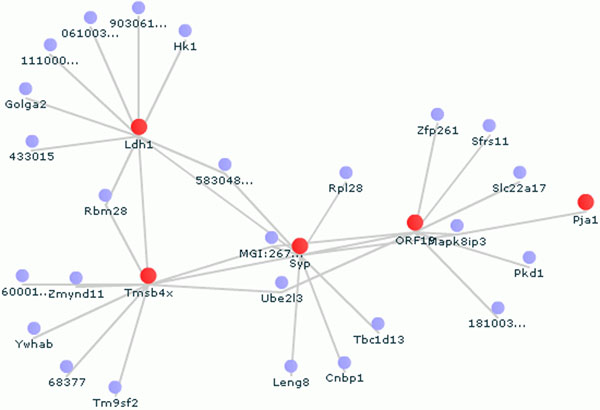
**Gene network linked by expression correlation** PubAnatomy can draw dynamic network graphs for genes that are highly correlated with a selected gene based on the Allen Brain Atlas 200 micron voxel gene expression data. The network graph is also expandable.

As a literature and data exploration tool, PubAnatomy allows users to search literature and provides different view and data tables for filtering and sense making. The UI has three major components: 1) graphic views on the main window provide data overview and starting points for data exploration; 2) tabulated data tabs in the bottom contain information relevant to the current view and selection, such as current brain structure, citation set, selected genes, etc.; 3) Tabs and menus on top of the main window are for selecting parameters and initiating analysis. The right panel contains search functions, user input and user history management. The following is a brief description of functions and data currently included in PubAnatomy:

1) BrainMap view for presenting related structural and functional information. When a user issue a keyword search, PubAnatomy will search literatures by using either a local search engines that we implemented for brain-related literature, or by calling the NCBI E-utilities web service on PubMed. The related citations will be mapped to brain structures using our pre-indexed tables. A coronal brain section with the largest number of PMID hit is than selected for the main window. Each colored region is a brain structure. Users can click on a structure to retrieve related Medline records. The color of labels of each structure indicates if there are citations mapped to the structure. When a different structure is selected, contents in the data tabs under the main window, such as Mesh profiling, related disease, In situ image mappings, and protein- protein interactions associated with genes in the related Medline records will be automatically updated.

2) Gene expression data display: Since the left and the right side of the mouse brain are symmetric, the BrainMap view can be used to visualize multiple data sources on the same brain image section. For example, a user can select a gene associated with a Medline record in the Gene Tab under the main window and choose to draw a gene expression heatmap based on Allen Brain Atlas 200 micron voxel gene expression data on the left brain while keeping the citation mapping on the right half of the map (Figure 2: Comt gene is selected) Such expression heatmap overlay on actual brain image allows researchers to easily select structures for further investigation based on the overview of gene expression across different annotated structures.

3) Brain circuits: The brain map can also display brain structures that are know to be connected to each other. Right clicking a brain structure will show a structure-specific context menu, and the user can choose to show a circuit that links current region to other brain regions.

4) Gene expression correlation network: PubAnatomy can draw dynamic network graphs for genes that are highly correlated with a selected gene based on the Allen Brain Atlas 200 micron voxel gene expression data (Figure 3). The network graph is also expandable. Users can right click on a gene node and choose to expand the network by including top ranked genes of the newly selected gene. Double clicking on a gene node will show information of edges related to this gene in the expression correlation table.

5) Data integration and mappings: Our solution integrates data from many sources and provides dynamic linkage among them. For example, the Mesh profiling tab presents MeSH terms that differentiate the current citation set from whole Medline corpus using a number of different criteria. The MiMI interaction tab shows genes known to be interacting with genes annotated with the current Medline citation set using information from Michigan Molecular Interactions (MiMI) database.

We have implemented a total of 9 data integration and analysis tabs, with each tab pulling information from multiple data sources for the current Medline citation set on- the-fly. Because of the flexible architecture and component-based design, PubAnatomy can easily accommodate new resources into the service layer and the UI. Since no data is embedded in the PubAnatomy client and PubAnatomy only requests information as needed, the program is very lightweight thus it can handle fairly large datasets with high efficiency. In addition, data tabs are linked to the content and selection of the main window: once the citation or gene set is changed, all associated tabs will automatically update.

6) Auxiliary tools: we have built charting tools (e.g. pie chart, line chart, etc) in PubAnatomy so that researchers can examine the values or distributions of current datasets without making extra effort to export the data to desktop tools. An image section selection tool is also provided to allow users to pin down a specific section of interest. Users can also customize the views, data tabs and context menu to fit their exploration process better.

### C. Interoperability with other applications

**Figure 4:**
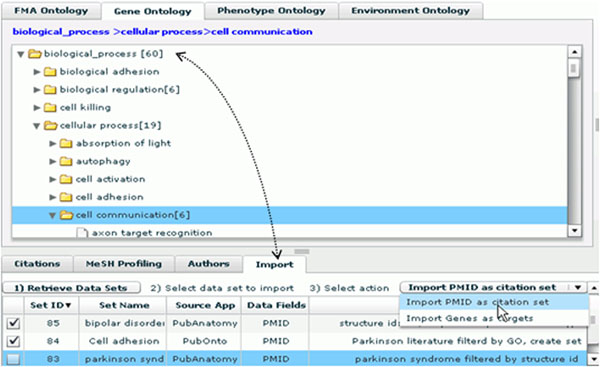
**Data sharing with other programs** PubAnatomy emphasize the interoperability by adopting the aforementioned data sharing and user management schema. It allows a users to export a dataset, name the dataset, record its parameters, write description and choose whether to share the dataset. Other applications or PubAnatomy per se can retrieve the dataset from the central database for additional analysis. As this figure demonstrates PubOnto, another program developed in our group, imports a set of PMIDs exported from PubAnatomy and maps it to Gene Ontology for identifying potential cellular and molecular processes related to a brain structure.

PubAnatomy emphasize the interoperability by adopting the aforementioned data sharing and user management schema. When a user login from PubAnatomy, he/she can export datasets, e.g. citation PMIDs, to a central database. He can name the dataset, record its parameters, write description and choose whether to share the dataset. Other applications or PubAnatomy itself can retrieve the dataset from the central database for additional analysis. As an example, Figure 4 demonstrates PubOnto, another program developed in our group, imports a set of PMIDs exported from PubAnatomy and maps it to Gene Ontology for identifying potential cellular and molecular processes related to a brain structure.

Similarly, PubAnatomy can import datasets generated by other programs as well. Such cross-application interoperability greatly enhances the ability to perform in- depth data mining by taking advantage of functionally complementary applications.

### D. Use case example

To illustrate how PubAnatomy can help the cross domain exploration of literature and data, we use a real world example of a researcher who seeks to uncover how any of the 20 genes found to be associated with CNV (copy number variant) sites in schizophrenic patients may be related to schizophrenia [22]. She queries PubAnatomy for “schizophrenia” and retrieves hundreds of Medline records mapped to different brain structures. She simultaneously sees other perspectives on these records, such as significantly occurring MeSH terms, genes and diseases associated with the records. She scans the PubAnatomy Gene Tab to identify genes associated with these Medline records and finds one from her list – Grin1. With a keystroke, she invokes PubAnatomy to paint this gene’s expression levels on the brain map image. From pseudo color expression level coding, the researcher sees immediately that Ammon’s Horn has high expressions of Grin1, an area that the expression of DISC1 gene (Disrupted-In-Schizophrenia 1) also concentrates [23]. With one mouse click on this region, the researcher now filters down to articles, diseases, and genes relevant only to this region (29 citations). She saves the citations for later and now seeks to uncover other types of relationships between the deleted genes and schizophrenia, as most of them did not show up as genes associated with the retrieved Medline records. She guesses that protein-protein relationships may connect the deletion genes (one gene list) and genes in the retrieved Medline records (another list). She clicks the "Find Path for All Genes" function in the right click menu of the Gene Tab, which supports finding potential protein-protein relationships, and then paste the 20 deletion gene list. Results show that FYN, which the researcher had not previously considered to be relevant, interacts with genes in both lists and was reported to be related to schizophrenia [24-28].

Though such quick explorations, the researcher has obtained some promising leads that are well worth detailed investigation. As the use case demonstrates, besides the fact that the researcher can efficiently find these leads, the integration of cross domain data and literature in PubAnatomy in fact presented multiple directions for exploration that will be hard to perform systematically for each possibility if the related data are distributed in different locations in the absence of a unified interface.

## Discussion

The purpose of the cross-domain data integration effort described here is to provide a more effective solution for understanding the biological significance of high throughput data.

The PubAnatomy prototype we developed is a novel solution that uses the mouse brain atlas as a framework for integrating literature, gene expression data and conceptual relationships from external databases.

Top on the list are the identification of important physiological processes, behavioral processes, and small molecules related to each brain structure. For physiological and behavioral processes, we choose to use UMLS concepts belonging to "Biologic Function" and "Behavior" semantic categories of the UMLS Semantic Network. Similar to our implementation for brain structure to disease associations, we will provide multiple ranking results for associating these concepts to individual brain structures. For integrating small molecule information, the terms for concept mapping will initially come from UMLS concepts belong to the "Chemical" semantic group, compounds and substances from PubChem, and the drug name list from Medline Plus. Our concept mapping solution provides highly efficient and straightforward data integration based on concept matching. However, this solution has an inherent shortcoming: context information for each concept is not automatically considered. In different contexts, the same text string may refer to different concepts. For example, the text string "cold" may refer to either the concept "cold in temperature" or the concept "symptom of catching a cold" depending on the context. To increase the rate of correct mappings for such ambiguous text strings, we plan to apply the context- based disambiguation approach used in our gene and genetic marker name recognition engines [18,19] .

We are also planning to add a functionality to compare the gene expression correlation network included in PubAnatomy with the protein-protein interaction network defined in the MiMI database [29,30]. Currently the gene expression correlations and protein interaction networks are displayed in separate applications (PubAnatomy and MiMI CytoScape plugin). The separate applications make it difficult to see relationships between gene expression correlations and protein interaction networks. A relatively simple solution is to make the MiMI CytoScape plugin capable of importing gene-gene relation tables from PubAnatomy. Then one could use CytoScape's built in network comparison function to identify highly correlated genes whose protein products directly interact with each other or can be linked in a protein-protein interaction network within a given distance. Such ability to compare networks derived from different data sets related to gene/protein or other types of concepts will greatly enhance researchers' capability to identify potentially interesting relationships for hypothesis development. The key to this enhancement is cross-application interoperability of the same type as we developed for PubAnatomy and PubOnto. We are actively working with our colleagues in the National Center for Integrative Biomedical Informatics (NCIBI) to achieve interoperability across all major applications, including the MiMI CytoScape plugin, developed at NCIBI.

Besides cross-application interoperability, it is essential to give researchers and third party developers the capability to extend PubAnatomy. Researchers and developers should be able to add new conceptual relationships, experimental data, data analysis functionality, and display functionality. A cross-domain data integration and exploration solution is not likely to be successful without participation from the target research community. No single group or company has the resources to integrate useful conceptual relationships and data from all related research areas. We are committed to generate detailed documentation describing the API for PubAnatomy before its formal public release around July 2009. We are looking forward to working with research groups interested in integrating mouse brain functional imaging data or higher resolution brain substructure data into the PubAnatomy system.

Lastly, although our current prototype is based on Allen Brain Atlas mouse brain structure annotation, the technical solutions we developed can be readily applied to annotated voxel data from any systems. Our main goal for the next phase is to integrate data from the Visible Human Projects [31,32]. We expect the integration of human anatomy data and the related functional and structural data from molecular to organism levels will greatly facilitate the understanding of high throughput data and the development of novel hypotheses.

## Competing interests

The authors declare that they have no competing financial interests.

## Authors' contributions

W. Xuan is the main developer of the PubAnatomy project. M. Dai developed the free text to brain region mapping engine used in this project. All authors participated in the design, evaluation and improvement of the PubAnatomy with main efforts from W. Xuan, B. Mirel and F. Meng. All authors contributed to and approved the manuscript.
